# Survival outcomes of patients with metastatic non-small cell lung cancer receiving chemotherapy or immunotherapy as first-line in a real-life setting

**DOI:** 10.1038/s41598-023-36623-1

**Published:** 2023-06-13

**Authors:** Yaniss Belaroussi, Fanny Bouteiller, Carine Bellera, David Pasquier, Maurice Perol, Didier Debieuvre, Thomas Filleron, Nicolas Girard, Roland Schott, Simone Mathoulin-Pélissier, Anne-Laure Martin, Sophie Cousin

**Affiliations:** 1 UMR 1219, Univ. Bordeaux, Bordeaux Population Health Research Center, Epicene Team, 33000 Bordeaux, France; 2 Inserm CIC1401, Clinical and Epidemiological Research Unit, Institut Bergonié, Comprehensive Cancer Center, 33000 Bordeaux, France; 3 Radiotherapy Department, Centre Oscar Lambret, 59000 Lille, France; 4 Medical Oncology Department, Centre Léon Bérard, 69373 Lyon, France; 5 Chest Disease Department, GHRMSA, 68100 Mulhouse, France; 6 Biostatistic and Health Data Science Unit, Institut Claudius Régaud IUTC-O, 31300 Toulouse, France; 7 Medical Oncology Department, Institut du Thorax Curie-Montsouris, 75014 Paris, France; 8 Medical Oncology Department, Institut de Cancérologie Strasbourg Europe, 67200 Strasbourg, France; 9 Health Data and Partnership Department, Unicancer, 75654 Paris, France; 10 Early Phase Trials Unit, Department of Medical Oncology, Institut Bergonié, Comprehensive Cancer Center, 229 Cours de L’Argonne, 33000 Bordeaux, France

**Keywords:** Cancer, Lung cancer, Non-small-cell lung cancer

## Abstract

Treatment of metastatic non-small cell lung cancer (mNSCLC) has been modified due to the development of immunotherapy. We assessed survival outcomes (overall [OS] and progression-free [rwPFS] survivals, time-to-next-treatment [TNT]) in mNSCLC patients after first-line immunotherapy and chemotherapy in real-life settings. Association between rwPFS and TNT, two candidate surrogate endpoints (SE), with OS was assessed. This retrospective multi-center study uses data from patients included in the Epidemio-Strategy Medico-Economic program with mNSCLC over 2015–2019. The impact of treatment on rwPFS/OS was evaluated with Cox models. Individual-level associations between SE and OS were estimated with an iterative multiple imputation approach and joint survival models. The population included 5294 patients (63 years median age). Median OS in immunotherapy group was 16.4 months (95%CI [14.1–NR]) and was higher than in chemotherapy group (11.6 months; 95%CI [11.0–12.2]). Improved OS was observed for the immunotherapy group after 3 months for subjects with performance status 0–1 (HR = 0.59; 95%CI [0.42–0.83], p < 0.01). The associations between rwPFS and TNT with OS were close ($\uptau$=0.57). Results emphasized a survival improvement with immunotherapy for patients in good health condition. There was moderate evidence of individual-level association between candidate SE and OS.

## Introduction

Metastatic non-small cell lung cancer (mNSCLC) is associated with poor prognosis, with 4% 5-year net survival rate^1^. Treatment has been modified due to the recent development of immune checkpoint inhibitors (ICIs). Randomized clinical trials (RCT) have demonstrated an improvement of overall survival (OS) of mNSCLC patients with first-line including immunotherapy as compared to chemotherapy^2–4^. These results led to an actualization of the European Society for Medical Oncology (ESMO) guidelines since 2017 recommending pembrolizumab as first treatment after the diagnosis of mNSCLC with Program Death-Ligand 1 (PD-L1) expression of 50% or more for patients with performance status between 0 and 2^4^. Moreover, chemotherapy and pembrolizumab combination as first-line treatment is recommended for patients regardless of PD-L1 expression, since 2018 for non-squamous mNSCLC^5–8^, and since 2019 for squamous mNSCLC^5,9,10^. In the first-line setting, monotherapy with pembrolizumab has been reimbursed for patients with PD-L1 expression ≥ 50% since 05/2017 in France. Pembrolizumab in combination with chemotherapy has been approved for patients with non-squamous histology since 11/2019 in France, and for patients with squamous histology since March 2020. Although RCT have strong internal validity, they suffer from restrictive eligibility criteria which limits generalizability of results to real-life settings.

Importance of alternative efficacy endpoints other than OS has grown in oncology^11–13^. PFS is commonly used as it is less likely to be affected by subsequent lines of treatment and is observed earlier than OS. The use of such an alternative endpoint relies on the hypothesis that it can adequately replace, i.e. be a valid surrogate endpoint (SE) for OS. The validation of an SE is a major issue relying on a meta-analysis of RCT. While PFS relies solely on disease progression evaluation, time to next treatment (TNT) is defined as the time to initiation of a new treatment after failure of the previous one. TNT thus reflects the clinical decision-making process including concepts of efficacy and toxicity. It has been associated with OS in several cancer subtypes such as sarcoma, myeloma, breast cancer, colon cancer, and prostate cancer^14–18^. In mNSCLC, there is to date, no valid surrogate endpoint and PFS and TNT thus still deserve to be investigated^19^.

Real world data (RWD) are defined as observational data from other sources than RCT such as electronic medical records, registries, insurance claims, pharmacy records, death certificates and other patient-generated data. Large real-world datasets thus present a unique opportunity to investigate treatment patterns and survival outcomes in real-life settings, as well as the individual-level association between candidate SE and OS, specifically for candidate endpoints not commonly reported in RCT, or with few RCT available to conduct a meta-analysis.

With the recent emergence of immune checkpoint inhibitors (ICIs), the objectives of this study were thus threefold. First, we investigated real-world (rw) patterns of care and survival outcomes (OS and rwPFS) in patients treated with either chemotherapy or ICIs. Second, prognostic factors for OS were assessed, including treatment (ICIs vs chemotherapy). Finally, we investigated the association between rwPFS and TNT, two candidates SE, with OS.

## Patients and methods

### Study design

This is a retrospective, observational, multicenter study using real-world data from routine care of mNSCLC patients treated in first intention with chemotherapy and/or anti PD1 inhibitor in French care centers.

### Data source

Data comes from the Epidemio-Strategy and Medical Economics (ESME) data platform for advanced and metastatic lung cancer (AMLC), a French national registry collecting and centralizing comprehensive real-life individual data on cancer management from a network 37 referring establishments (private non-profit Comprehensive Cancer Centers, University or General Hospitals) specializing in treating cancer. Methods were carried out in accordance with relevant guidelines and regulations. The ESME AMLC database (NCT03848052) was authorized by the French data protection authority in 2017 and focuses on adult patients with locally advanced or metastatic lung cancer who were diagnosed or initiated treatment from 2015. Informed consent was obtained from all participants and/or their legal guardians. Data are compiled from patient’s electronic medical record, inpatient hospitalization records and pharmacy records. Data are retrospectively collected using a well-structured electronic data collection tool approach by trained technicians on-site. Collected data includes patient demographic characteristics, pathology, outcomes and treatment patterns. All research protocols were approved by Unicancer. Data extraction took place on September 3rd, 2019.

### Study population

The selected population had a stage IV NSCLC treated in first line by ICIs or chemotherapy between January 2015 and January 2019. Participants with an ALK translocation or an EGFR mutation, and those who received first-line targeted therapy were excluded. Patients with antiangiogenic therapies were retained as these treatments do not target a specific oncogenic addiction.

For the analysis of survival outcomes, patients were compared according to their first-line treatment: chemotherapy or immunotherapy (Atezolizumab, Avelumab, Durvalumab, Ipilimumab, Nivolumab, Pembrolizumab or Tremelimumab) as single agent. For the analysis of candidate SE, the patients treated with a chemotherapy-ICI combination were included.

### Outcomes measures

Primary endpoint was OS, defined as the time between initiation of the first treatment for the mNSCLC and death. rwPFS was defined as the time between initiation of the first treatment and first disease progression assessed by treating physician or death. The date of the first progression was the date of the diagnosis of first event occurring after the start of treatment among: progression at regional site of the primary tumor or site of lymph nodes; a new metastasis; progression of a known metastasis; end of treatment due to the progression of the disease; death. TNT was defined as the time between initiation of the first treatment and the date of initiation of a second line of treatment or death. Discontinuation of a line of treatment may be due to several reasons: disease progression, treatment toxicity, or patient choice. Thus, the first line of treatment was defined by including all the treatment drugs received within 30 days after the start date of the first treatment. Any new drug received more than 30 days after this date was considered as the start of a second line treatment. This definition is conformed to the course of concomitant treatment protocols starting within 30 days (when there is combination of chemotherapy and ICIs).

### Statistical analysis

We described the characteristics the study population according to first-line therapy; we used counts and proportions for qualitative variables, and means, medians, interquartile range and standard deviations for quantitative variables. For survival outcomes, the date of initiation of the first-line therapy was chosen as the date of origin. The censoring date was the most recent date of last news. Survival data were described using Kaplan–Meier survival curves. Median survival times were reported with respective 95% confidence intervals (95%CI). Median follow-up time was estimated using the reverse Kaplan Meier method^20^.

We relied on Cox proportional hazards (PH) models for the assessment of the prognostic factors, including treatment, for rwPFS and OS. Hazard ratios (HR) and 95%CI were reported. PH assumption was explored graphically using a log(− log(S(t)) function plot.

Variable selection was performed using a directed acyclic graph (DAG) for adjustment to estimate the total effect of the first-line treatment on survival^21,22^. Candidate prognostic variables investigated were specific to the tumor (histological type defined as squamous or non-squamous, M stage categorized as Mx, M1a, M1b, M1c, metastasis localization described as in contralateral lung, pleural fluid, bone, liver, brain and nervous system, metastatic nodes, adrenal glands and other localization and PD-L1, the patient (age, ECOG performance status [PS], medical history and comorbidities as chronic obstructive pulmonary disease (COPD), autoimmune disease, asbestosis, tuberculosis, diabetes mellitus, kidney failure, heart failure, high blood pressure, history of other cancer and family history of lung or pleural cancer, smoking status categorized as active, former or non-smoker), and the center (we evaluated the participation to ESME database by center and by year and proportion of ICIs administration to describe center’s treatment administration).

PD-L1 expression was discussed as a prognostic factor and considered in subgroups analyses for subjects with available data.

To assess the robustness of the models, we conducted three distinct sensitivity analyses : (i) multiple imputation analysis of the missing data to adapt the analysis strategy according to the missing data mechanism; (ii) propensity score with reverse weighting to take into account the variability of receiving treatment depending on characteristics ; (iii) propensity score with matching^23,24^.

We investigated the association between the two candidates SE (rwPFS and TNT) and OS. In the context of a meta-analysis, validating surrogacy requires a strong correlation between the candidate SE and the final endpoint (individual-level association) and between the treatment effects observed on the candidate SE and the final endpoint (trial-level association). Here, we assessed the trial-level association using the center as the trial. As few patients were treated with ICIs for some centers, all patients treated with ICIs (monotherapy or combination) were kept for this exploratory analysis. We first relied on an iterative multiple imputation (IMI) approach to estimate the individual-level associations using the Waerden coefficient of correlation *r̂W*, similar to the usual rank-correlation of Spearman ρ^25^. Next, we applied a joint frailty model which allowed us to estimate simultaneously the individual- and trial(center)-level associations^26,27^. This yields an estimate of the Kendall τ coefficient, for the individual-level association, as well as a coefficient of determination (*R*^2^) for the center-level association. In this second strategy, the individual-level association was investigated including either a Gumbel Copula parameter or a random effect^27^. Coefficients of correlations range from − 1 to 1, with values close to 1 indicating strong positives correlations. We also estimated the Surrogate Threshold Effect (STE), i.e. the minimum treatment effect on the SE necessary to predict a significant effect on the true endpoint.

Statistical analyses were performed with R version 4.0.0. and SAS version 9.4. software.

## Results

ESME AMLC database included 21,169 patients treated for AMLC between January 1st, 2015 and January 31st, 2019. Men represented 66.4% of the population and women counted for 33.6% of the population. Median age at diagnosis of mNSCLC was 64 years.

**Figure 1:**
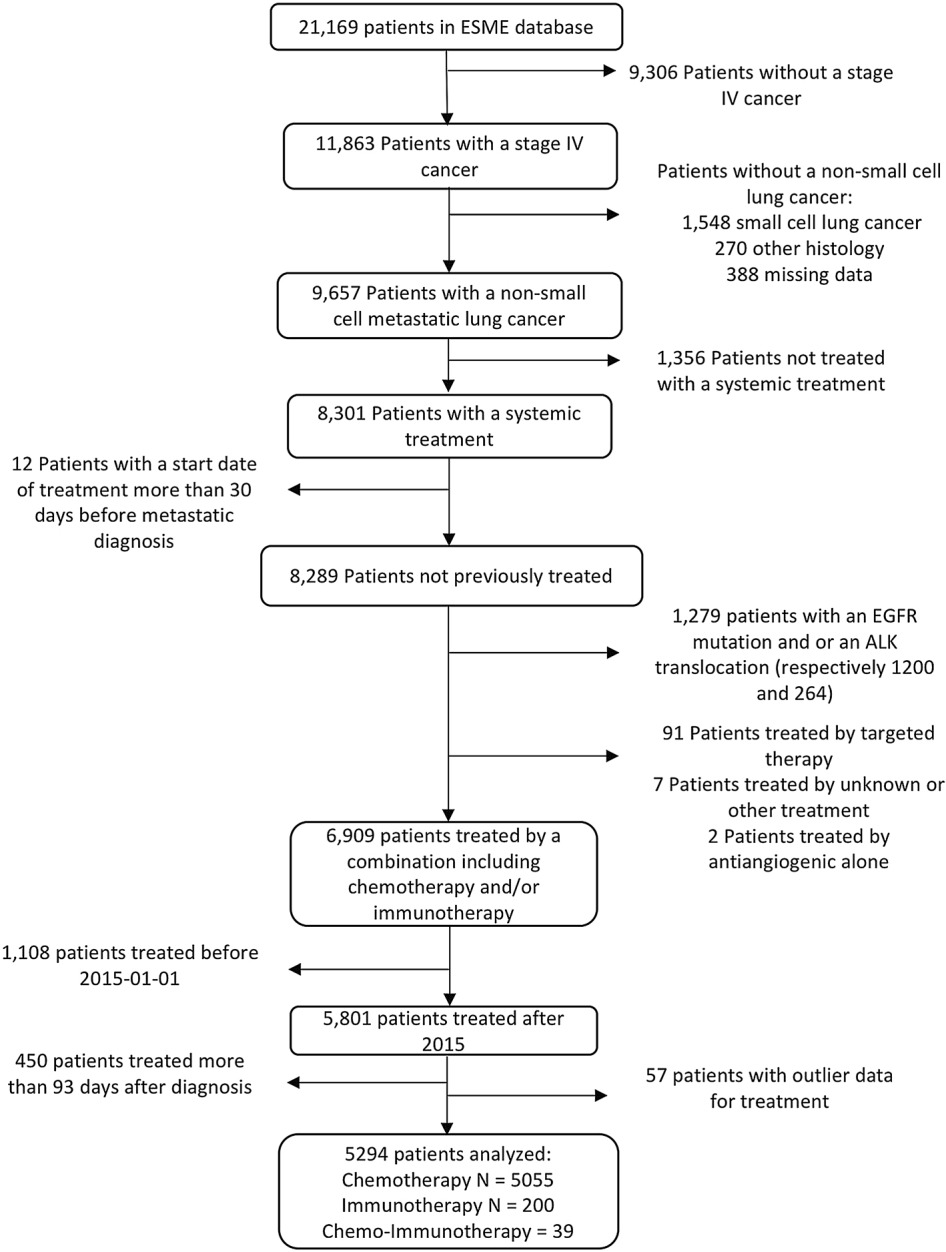
Flow-chart illustrating selection process for patients with metastatic lung cancer, ESME AMLC database, France, 2015–2019.

A total of 14,260 subjects were excluded due to absence of mNSCLC cancer, absence of systemic treatment received, or presence of EGFR mutation or ALK translocation. We excluded another 445 patients because of a long delay between metastatic diagnosis and treatment initiation (more than 3 months), and 57 patients because of incoherent data according to treatment protocol. Finally, only patients treated since 2015 were kept for the analysis, in order to account for the recent development of ICIs. Thus, 5294 individuals were included in the study: 5055 were treated with chemotherapy, 200 were treated with ICIs and 39 were treated with a combination of both (Fig. 1).

For the final set of analyzed patients, mean age at diagnosis of metastatic disease was 62.4 years, men represented 67.8% of the population. More than 90% of the population had a smoking history (active smokers and former smokers represented respectively 38.0% and 52.3%) however only 13.1% had a chronic obstructive pulmonary disease (COPD) diagnosis mentioned. Almost half of the patients had medical history (44.8%). Cardiovascular disease (heart failure and high blood pressure) was the most common associated disease and concerned one third of the overall population. About 60% of the population presented a Performance Status (PS) of 0–1.

Main histological type was non-squamous lung cancer (83.3%). The most frequent metastatic status was M1c. The M status was undetermined for 782 patients. The most frequent metastatic sites were bone (43.9%) and brain/nervous system localization (34.2%) (Table 1).

**Table 1:** Characteristics of the included population according to first-line therapy, ESME AMLC database, France, 2015–2019.

	All (N = 5294)	Chemotherapy (N = 5055)	Immune checkpoint Inhibitors (N = 200)	Chemo-immunotherapy (N = 39)	Missing data
N (%) or median (interquartile range)					
Patients characteristics					
Sex					
Male	3588 (67.8)	3420 (67.7)	136 (68.0)	32 (82.1)	0
Female	1706 (32.2)	1635 (32.3)	64 (32.0)	7 (17.9)	
Age at metastatic diagnosis	63 (56–69)	63 (56–69)	63 (57–70)	62 (56.5–68)	0
Comorbidities					
Smoking status					
Active smoker	2013 (38.0)	1921 (39.5)	74 (38.5)	18 (46.2)	205 (3.9)
Former smoker	2770 (52.3)	2643 (54.4)	108 (56.2)	19 (48.7)	
Non-smoker	306 (5.8)	294 (6.1)	10 (5.2)	2 (5.1)	
Medical history	2373 (44.8)	2262 (47.1)	92 (46.5)	19 (50.0)	252 (4.8)
COPD	691 (13.1)	652 (12.9)	34 (17.0)	5 (12.8)	
Autoimmune disease	112 (2.1)	109 (2.2)	3 (1.5)	0 (0.0)	
Asbestosis	5 (0.1)	5 (0.1)	0 (0.0)	0 (0.0)	
Tuberculosis	80 (1.5)	77 (1.5)	3 (1.5)	0 (0.0)	
Diabetes mellitus	632(11.9)	603 (11.9)	24 (12.0)	5 (12.8)	
Kidney failure	85 (1.6)	82 (1.6)	1 (0.5)	2 (5.1)	
Heart failure	104 (2.0)	101 (2.0)	3 (1.5)	0 (0.0)	
High blood pressure	1654 (31.2)	1582 (31.3)	57 (28.5)	15 (38.5)	
Other cancer	787 (14.9)	750 (15.6)	33 (16.8)	4 (10.8)	257 (4.9)
Family history of lung or pleural cancer	380 (7.2)	368 (17.1)	11 (11.7)	1 (8.3)	3041 (57.4)
PS ECOG					
0	1052 (19.9)	992 (22.9)	46 (25.5)	14 (43.8)	753 (14.2)
1	2127 (40.2)	2024 (46.7)	90 (50.0)	13 (40.6)	
2	966 (18.2)	933 (21.6)	31 (17.2)	2 (6.2)	
3	345 (6.5)	332 (7.7)	10 (5.6)	3 (9.4)	
4	51 (1.0)	48 (1.1)	3 (1.7)	0 (0.0	
Tumor characteristics					
Histological type					
Squamous	883 (16.7)	838 (16.6)	41 (20.5)	4 (10.3)	0
Non-squamous	4411 (83.3)	4217 (83.4)	159 (79.5)	35 (89.7)	
M stage					
Mx	786 (14.8)	753 (14.9)	29 (14.5)	4 (10.3)	0
M1a	311 (5.9)	297 (5.9)	12 (6.0)	2 (5.1)	
M1b	1424 (26.9)	1361 (26.9)	46 (23.0)	17 (43.6)	
M1c	2773 (52.5)	2644 (52.3)	113 (56.5)	16 (41.0)	
Metastasis localization					
Contralateral lung	1350 (25.5)	1290 (25.5)	49 (24.5)	11 (28.2)	
Pleural fluid	263 (5.0)	251 (5.0)	8 (4.0)	4 (10.3)	
Bone	2322 (43.9)	2216 (43.8)	90 (45.0)	16 (41.0)	
Liver	878 (16.6)	838 (16.6)	37 (18.5)	3 (7.7)	
Brain and nervous system	1795 (34.2)	1739 (34.4)	47 (23.5)	9 (23.1)	
Metastatic nodes	846 (16.0)	802 (15.9)	39 (19.5)	5 (12.8)	
Adrenal glands	1374 (26.0)	1294 (25.6)	68 (34.0)	12 (30.8)	
Other	130! (24.8)	1248 (24.7)	53 (26.5)	7 (17.9)	
PDL1 expression					
< 50% or negative	1053 (19.9)	1019 (20.2)	24 (12.0)	10 (25.6)	3831 (72.9)
≥ 50%	385 (7.3)	248 (4.9)	133 (66.5)	4 (10.3)	
Unknown	3856 (72.8)	3788 (74.9)	43 (21.5)	25 (64.1)	

*ECOG PS* Eastern cooperative oncology group, performance status, *COPD* chronic obstructive pulmonary disease.

Distributions for sex and smoking status were similar in both chemotherapy and ICIs groups, with few non-smokers. Median age at metastatic diagnosis was similar in both groups (63 years). Distribution of COPD, diabetes mellitus, cardiovascular diseases, tuberculosis and other cancers were rather comparable in both groups. Family history of lung or pleural cancer proportion was higher in chemotherapy group. PS 2-3-4 was more frequent in chemotherapy group (30.4% vs. 24.5%). Brain and nervous system metastases were more frequent in chemotherapy group than in the ICIs group which presented more adrenal glands metastases.

PD-L1 expression suffered from missing data (72.9% missing data). Among the ICIs group, data was available for 157 patients (78.5%) and PD-L1 expression was 50% or higher in 133 patients (84.7% of the patients with available data). In chemotherapy group, 25.1% of the patients had PD-L1 data available and among them, 19.6% had a PD-L1 expression of 50% or higher. Guidelines recommended administration of ICIs as single agent for patients with ECOG PS 0–1 and PD-L1 expression ≥ 50%. Of the 147 subjects treated by pembrolizumab with both known PD-L1 status and performance status, 82 (55.8%) received treatment as recommended by the guidelines. Indeed, 24 subjects had PD-L1 expression below 50% and were anyway treated with ICIs as well as 44 patients with poor PS. Only eight of those treated outside guidelines were included in a clinical trial.

Among the 5294 patients, median follow-up time was 22.1 months [11.6–32.6], ranging from 13.8 months (95% CI [9.1–17.8]) for the ICI group, to 22.9 months (95%CI [11.8–33.0]) for the chemotherapy group. Median OS was 11.8 months (95%CI [11.0–12.2]), median rwPFS was 4.0 months (95% CI [3.9–4.1]) and median TNT was 4.3 months (95% CI [4.1–4.4]).

**Figure 2:**
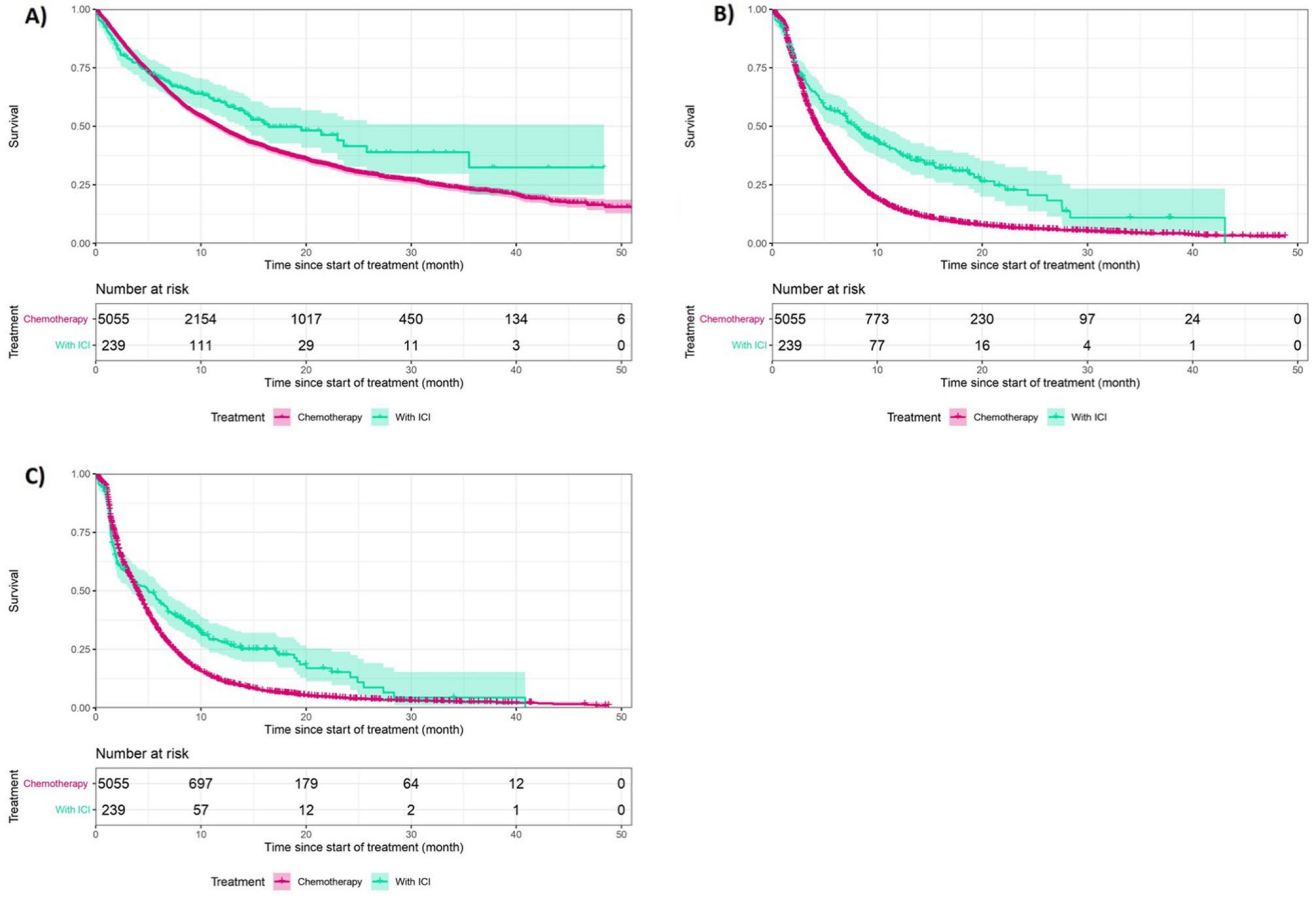
Overall Survival (**A**), Progression Free Survival (**B**) and Time to Next Treatment (**C**) according to treatment group, ESME AMLC database, France 2015–2019 (in pink: chemotherapy; in green: immune checkpoint inhibitors (ICIs)).

Median OS in the ICI group was 16.4 months (95% CI [14.1–NR]), higher than for the chemotherapy group (11.6 months; 95% CI [11.0–12.2]) (Fig. 2). Median rwPFS in the ICI group was 5.0 months (95% CI = [3.0–6.9]) whereas it was 4.0 months (95%CI [3.8–4.1]) in the chemotherapy group. Finally, median TNT was 4.3 months for the chemotherapy group, and 7.4 months for those who received ICIs. One-year survival rate was higher in the ICI group (60.8%; 95%CI [53.9–68.4] versus 48.9%; 95%CI [47.4–50.4]).

Concerning prognostic factors of OS (Table 2), two sets of adjustment variables were explored. Both models 1 and 2 included the following adjustment variables: age, sex, center participation, comorbidities, liver metastasis, M status, histology and performance status. Model 1 accounted further for the delay between diagnosis and treatment initiation. On the other hand, model 2 accounted for the year of treatment. Results with regards to the association between treatment and OS were similar for the two models. The evaluation of the proportionality of the risks conducted to a separation of the timeline at 3 months for both OS and rwPFS. Among subjects with PS 0–1, treatment had no significant effect on survival before 3 months whereas the instant risk of death after 3 months was about 40% significantly lower in ICI group (for model 1 and 2 respectively, HR = 0.59; 95%CI = (0.42 – 0.83), P = 0.003 and HR = 0.58; 95%CI (0.41–0.83), P = 0.002 (Table 2). Among subjects with PS 2-4, ICI was significantly associated with an increased instant risk of death of 125% before 3 months but no significant effect on survival after 3 months was found.

**Table 2:** Main survival analyses with complete data for the primary and the secondary outcomes according to first-line treatment, immune checkpoint inhibitors vs. chemotherapy (reference) (N = 4277), ESME AMLC Database, France 2015–2019.

	Overall survival			Progression-free survival		
	HR	95% CI	P-value	HR	95% CI	P-value
Model 1^a^: immune checkpoint inhibitors vs chemotherapy (ref)						
PS 0-1^c^						
Before 3 months	1.00	0.61–1.64	0.985	1.02	0.76–1.37	0.893
After 3 months	**0.59**	**0.42–0.83**	**0.003**	**0.46**	**0.34–0.61**	** < 0.001**
PS 2–3-4^c^						
Before 3 months	**2.28**	**1.17–4.47**	**0.016**	**1.65**	**1.02–2.67**	**0.040**
After 3 months	0.85	0.36–2.04	0.719	0.84	0.34–2.13	0.720
Model 2^b^: immune checkpoint inhibitors vs chemotherapy (ref)						
PS 0-1^c^						
Before 3 months	0.98	0.60–1.62	0.944	0.98	0.73–1.31	0.893
After 3 months	**0.58**	**0.41–0.83**	**0.002**	**0.44**	**0.33–0.59**	** < 0.001**
PS 2–3-4^c^						
Before 3 months	**2.25**	**1.15–4.39**	**0.018**	**1.63**	**1.01–2.63**	**0.047**
After 3 months	0.84	0.35–2.01	0.689	0.83	0.35–2.01	0.692

Significant results are in bold.

*Ref* reference, *95% CI* confidence interval of 95%.

^a^Model 1: adjustment on gender, age, performance status, metastases localization, center, comorbidities (kidney failure, cardiovascular disease, history of other cancer), histology, delay to treatment initiation.

^b^Model 2: adjustment on gender, age, performance status, metastases localization, center, comorbidities (kidney failure, cardiovascular disease, history of other cancer), histology, year of initiation.

^c^p-value for interaction treatment with performance status < 0.001.

Concerning rwPFS, conclusions of models 1 and 2 were equivalent (Table 2): among subjects with PS 0-1, treatment had no significant effect on progression-free survival before 3 months, however the instant risk of progression or death after 3 months was about 55% significantly lower in immunotherapy group; among subjects with performance status of 2 to 4, the treatment with ICIs was significantly associated with an increased instant risk of death of 63% (model 1) and 65% (model 2) before 3 months but no conclusive effect on survival after 3 months was found.

**Figure 3:**
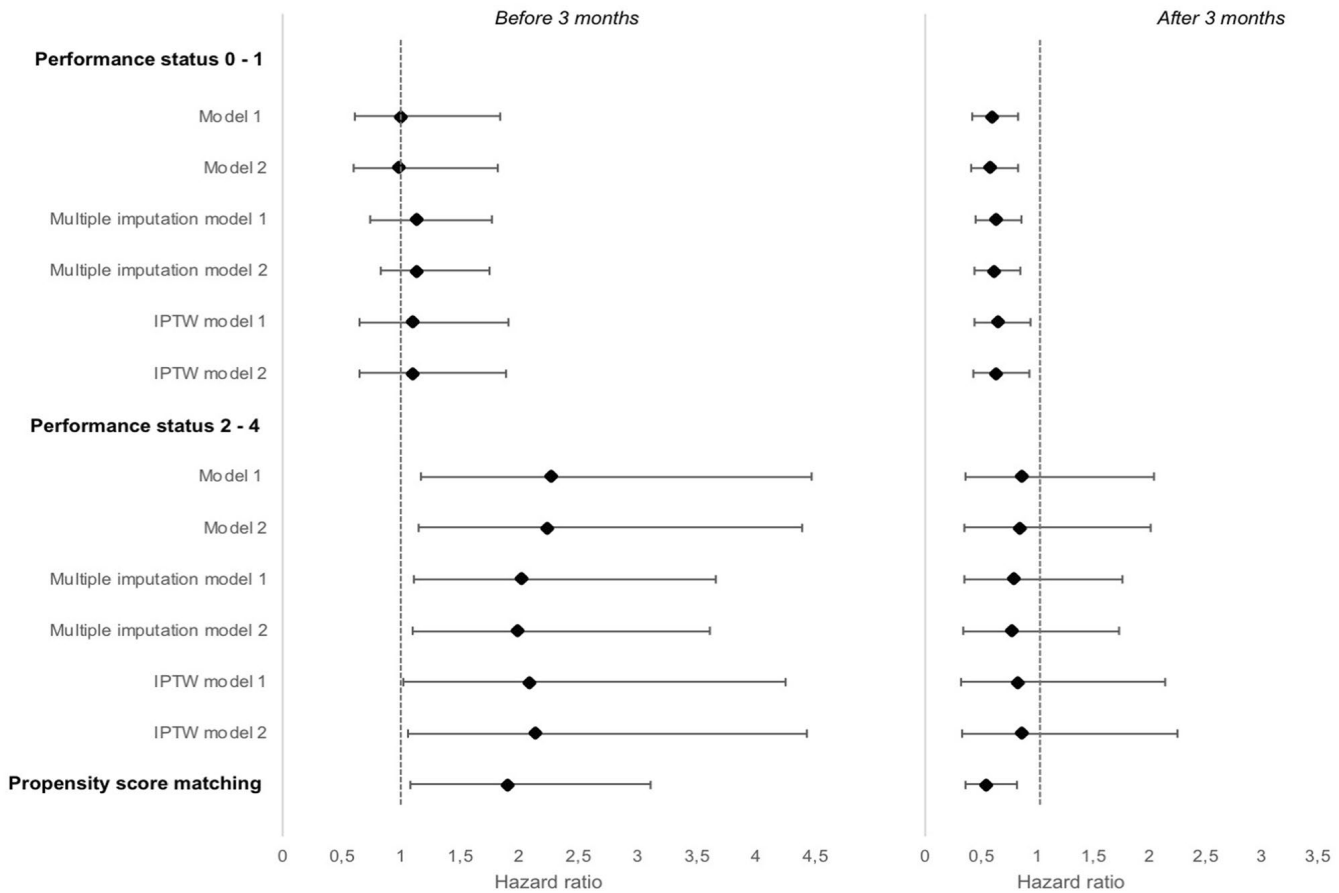
Results of sensitivity analyzes for overall survival. Hazard ratio for overall survival, immunotherapy vs chemotherapy (reference), ESME AMLC, France 2015–2019.

Sensitivity analyses yielded similar estimates than those obtained in the main analysis (Fig. 3). Results from stratified analyses on the PD-L1 expression were not statistically significant (Supplementary Material S1).

Concerning the associations between the candidate SE and OS, 13 centers were selected for this analysis to ensure convergence of the models (N = 3094). The estimated Waerden’s correlation between rwPFS and OS, as per IMI, was high with rW = 0.78 (95% CI [0.77–0.80]). With the joint frailty model approach, the individual-level association between OS and rwPFS estimated by Kendall's rate was moderate, with a maximum value of τ = 0.57 (0.54–0.61) reached for the joint model with Gumbel's copula (Table 3). Estimated coefficients for associations at the center-level (${\mathrm{R}}_{\mathrm{center}}^{2}$) were close to 1. Results were similar using joint frailty model. For the association between rwPFS and OS and, the joint model with Gumbel's copula had the highest estimate of the STE. This suggests that in a future center, with an effect of treatment on rwPFS corresponding to a hazard ratio of 0.91 maximum, it will be possible to predict a significant effect of treatment on OS.

**Table 3:** Evaluation of the individual level (Kendall's $\tau$) association and the center-level (R^2^) association between the surrogate endpoints and OS from different joint frailty models. ESME AMLC Database, France 2015–2019. (N = 3094).

Estimated parameters	Gumbel copula joint frailty model	Joint frailty model
rwPFS		
${\mathrm{HR}}_{\mathrm{Surrogate}}$	0.83 (0.54–1.29)	0.80 (0.53–1.21)
${\mathrm{HR}}_{\mathrm{OS}}$	0.62 (0.35–1.09)	0.77 (0.52–1.13)
$\mathrm{Kendall}$ $\uptau$ (95% CI)	0.57 (0.54–0.61)	0.44 (0.43–0.46)
${\mathrm{R}}_{\mathrm{center}}^{2}$(95% CI)	1.00 (0.91–1.09)	1.00 (0.86–1.14)
STE (HR)	[0.72–0.91*]	[0.35–0.87*]
TNT		
${\mathrm{HR}}_{\mathrm{Surrogate}}$	0.56 (0.34–0.92)	0.51 (0.33–0.77)
${\mathrm{HR}}_{\mathrm{OS}}$	0.67 (0.38–1.18)	0.79 (0.51–1.22)
$\mathrm{Kendall \tau }$(95% CI)	0.57 (0.53–0.60)	0.54 (0.53–0.56)
${\mathrm{R}}_{\mathrm{center}}^{2}$(95% CI)	1.00 (1.00–1.00)	1.00 (0.94–1.06)
STE (HR)	[0.31–0.63*]	[0.44–0.53*]

*95% CI* 95% confidence interval, *STE* Surrogate threshold effect, *HR* hazard ratio.

* represents the STE, the smallest effect of treatment (in HR) on rwPFS which allow to predict a significant effect of treatment on OS.

The estimated correlation between TNT and OS, as per IMI, was 0.76 (95% CI [0.74–0.77]). With the second approach, the individual-level association between TNT and OS was moderate, with a maximum value of τ = 0.57 (0.53–0.60) also reached for the joint frailty model with Gumbel's copula. Estimated coefficients for associations at the center-level (${\mathrm{R}}_{\mathrm{center}}^{2}$) were close to 1. The estimated STE was 0.63.

## Discussion

We aimed to compare chemotherapy and ICIs treatment as first-line treatment for mNSCLC based on real-life data. ESME AMLC database included 21,169 consecutive patients with a similar sex ratio than the overall population of patients with lung cancer described by the French National Institute of Cancer in 2018^28^ (66.4% of male vs 67.4% of male). Moreover, the median age at diagnosis is quite the same (63 years for female in ESME AMLC database vs 67 years in National institute for Cancer analyses, and 65 years for male in both databases).

Our analyses relied on 5294 patients presenting a mNSCLC diagnosed between January 2015 and January 2019. Their characteristics were very consistent with world demographic data^29^: they were mainly male, median age of 63 years old, with a history of smoking for more than 90% of them and the most frequent histologic diagnosis was non-squamous cell. Most of them were treated with chemotherapy and only 200 subjects were treated with ICIs and 39 with both chemotherapy and ICIs.

Descriptive data suggested that patients treated with ICI may have better outcomes, although survival curves crossed-over for OS, rwPFS and TNT (Fig. 2). Our adjusted analyses concluded that after 3 months, ICI had better results on survival than chemotherapy for patients in good health (PS 0-1) (for model 1 and 2 respectively, HR = 0.59; 95%CI 0.42–0.83, P = 0.003 and HR = 0.58; 95% CI 0.41–0.83). However, ICI had poor results before 3 months for subjects who are in generally poor health (PS 2-4). Sensitivity analyses performed, i.e. analyses with multiple imputation performed to increase the power of the study and analyses with propensity score methods to consider confounding in another way, strengthened these conclusions.

This study brings many perspectives on real-life data: results of clinical trials can be transposed into real-life despite restrictive selection criteria, as the studied population included patients with brain metastasis and subjects with PS > 1. However, Beaulieu-Jones et al.^30^ identified some limitations of real-life data: (1) unobserved confounding factors which can influence the doctor's decision and lead to an indication bias (for example, patient's request, inclusion in a trial or unequal access to treatment); (2) evolution of medicine over time, (3) the possible underestimation of adverse effects due to a less frequent follow-up than clinical trials; (4) a lack of completeness of the data and a need for harmonization of data to strengthen accuracy. Some patients with PDL1 < 50% received ICI treatment instead of chemotherapy which could be explained by an indication bias: the choice of the treatment is left to the appreciation of the physician in charge of the patient. This is why we have structured our main analysis using a directed acyclic diagram, in order to limit confounding and indication biases. Moreover, our three sensitivity analyses corroborated our findings by highlighting the robustness of our results. Thus, our results suggest that ICI treatment in first-line treatment for metastatic lung cancer has a benefit on overall survival for patients in good general condition, beyond 3 months of treatment. ICI treatment should be avoided for patients with poor health condition. However, it would be interesting to conduct a similar study with a larger group of subjects treated with ICI. In addition, our study highlighted the need to understand the role played by PD-L1 expression in the efficacy of treatment. On the one hand, it would be necessary to focus on the independent causal association between PD-L1 expression and survival. On the other hand, it would be relevant to assess whether there is a threshold for PD-L1 expression for the efficacy of immunotherapy, and compare findings between controlled trials and real-life settings.

Regarding candidate SE, individual association rwPFS/OS and TNT/OS were moderate. Regarding the association considering treatment effect, the association was strong between OS and each SE: estimated coefficient of determination was close to 1 in each joint model. Of note, we relied on two approaches to estimate associations between two survival criteria. Both approaches used are conceptually different, thus leading to sometimes divergent results. Divergences can first be explained by the fact that one tries to approximate a Spearman's ρ, while the other estimates a Kendall τ. Moreover, with IMI method, coefficients can be “increased” by considering data right-censored by multiple imputations. First approach is similar to the latest studies carried out by studying a direct relationship without taking into account the correlation between treatment effects^11–13^. In this direction, results greater than 0.70 are similar with those found by Khozin et al.^12^. Moreover, while this approach estimates a simple correlation, the second method estimates a residual dependence between two individuals, after considering dependence between patient from the same center, and dependence between those who received the same treatment in the same center. The second approach therefore seems more suited to our data. Overall, this exploratory analysis does not support strong evidence of associations between rwPFS/TNT and OS. As the validation of SE relies on meta-analysis of RCT, one must thus wait for the publication of ICI trials to confirm our preliminary findings in this real-life setting.

Our real-world data set lacked systematic PD-L1 expression data to evaluate if benefit from ICI was restricted to PD-L1 expression ≥ 50% only, as it has been proven in RCT. As expertise and handling of ICI spread with years, no doubt that PD-L1 expression would be a routine assessment. Due to the necessity of PD-L1 expression determination standardization and its late implementation in routine practice, it could be of interest to conduct the same analysis with a more recent population, recorded from January, 1st 2019 since now to collect robust data regarding PD-L1 expression status. Then, in one hand, it would be interesting to study the independent association between PD-L1 expression and death and, in the other hand, to evaluate the benefit of the ICI treatment according the PD-L1 rates.

To the best of our knowledge, there is no published evidence regarding the comparison ICI treatment vs chemotherapy as first-line treatment for previously untreated patients with mNSCLC. We retrieved eleven articles studying real-life outcomes for patients treated by immunotherapy for mNSCLC^12,31–40^. Seven of them concerned immunotherapy as first-line treatment; characteristics of these studies and characteristics of the included patients are presented in Supplementary Material S3. Median OS of the immunotherapy group from our study is similar to those found in these three studies. Both studies of Khozin reported a lower median OS than our study, probably because these cohorts included less than 25% of patients naïve from any treatment before immunotherapy administration, i.e. disease was probably more severe. Nevertheless, median OS in Ksienski et al. study was the highest although second-line and more therapies were included. For instance, median OS of approximately 19 months in both cohorts in Velcheti et al. study was slightly higher than those in immunotherapy group. Besides, median rwPFS in immunotherapy group of 5.0 months was shorter than those in Velcheti et al. study (6.8 months). However, Velcheti et al. study selected only patients with ECOG PS 0 or 1 and PD-L1 expression ≥ 50%.

In conclusion, our study focused on a well-representative national population to provide first valid results in favor of benefits of ICIs as first-line treatment for metastatic lung cancer after 3 months for patients with ECOG PS 0-1, as found in clinical trials. These are first real-world results and need to be completed with others studies to integrate larger population and more information (i.e. PD-L1 expression).

## Supplementary Information

Supplementary Tables.

## Data Availability

Any request of data sharing should be sent to Unicancer. The central coordination team of Unicancer and the ESME Strategic Committee and Scientific Committee manage the ESME database.

## Abbreviations

AMLC: Advanced and metastatic lung cancer
COPD: Chronic obstructive pulmonary disease
DAG: Directed acyclic graph
ESME: Epidemio-strategy medico-economic
ESMO: European society for medical oncology
ICIs: Immune checkpoint inhibitors
mNSCLC: Metastatic non-small cell lung cancer
OS: Overall survival
PD-L1: Program death-ligand 1
RCT: Randomized clinical trial
*r̂**W*: Waerden coefficient of correlation
RWD: Real-world data
rwPFS: Real-world progression free survival
SE: Surrogate endpoints
TNT: Time-to-next treatment
