# Supplementary material for: Survival outcomes of patients with metastatic non-small cell lung cancer receiving chemotherapy or immunotherapy as first-line in a real-life setting
Source: Sci Rep. 2023 Jun 13;13:9584. doi: 10.1038/s41598-023-36623-1 (PMC10264352; doi:10.1038/s41598-023-36623-1)
Supplement: Supplementary file 1 — Supplementary Tables. [file 41598_2023_36623_MOESM1_ESM.docx]

| Supplementary material S1. Sensitivity analyses for the primary and the secondary outcomes according to first-line treatment with stratification on PD-L1 expression, immunotherapy vs chemotherapy (reference), | | | | | | | |
| --- | --- | --- | --- | --- | --- | --- | --- |
|  | Overall survival | | |  | Progression-free survival | | |
|  | HR | 95%CI | *P-value* |  | HR | 95%CI | *P-value* |
|  | | | | | | | |
| ***Patients with PDL1 expression* ≥*50% (N=381)*** | | | | | | | |
|  |  |  |  |  |  |  |  |
| **Model 1^b^: Immunotherapy vs chemotherapy (ref)** |  |  |  |  |  |  |  |
| PS 0-1 |  |  |  |  |  |  |  |
| Before 3 months | **2.76** | **1.01 – 7.53** | **0.048** |  | 0.84 | 0.52 – 1.34 | 0.456 |
| After 3 months | 0.93 | 0.34 – 2.51 | 0.881 |  | **0.56** | **0.36 – 0.87** | **0.009** |
| PS 2-3-4 |  |  |  |  |  |  |  |
| Before 3 months | 1.77 | 0.32 – 9.97 | 0.517 |  | 1.90 | 0.97 – 3.73 | 0.060 |
| After 3 months | NA | NA | NA |  | 1.03 | 0.37 – 2.87 | 0.953 |
|  |  |  |  |  |  |  |  |
| **Model 2^b^: Immunotherapy vs chemotherapy (ref)** |  |  |  |  |  |  |  |
| PS 0-1 |  |  |  |  |  |  |  |
| Before 3 months | 2.51 | 0.92 – 6.87 | 0.073 |  | 0.66 | 0.41 – 1.06 | 0.088 |
| After 3 months | 0.86 | 0.32 – 2.33 | 0.769 |  | **0.39** | **0.25 – 0.62** | **<0.001** |
| PS 2-3-4 |  |  |  |  |  |  |  |
| Before 3 months | 1.64 | 0.29 – 9.17 | 0.576 |  | **2.21** | **1.12 – 4.35** | **0.022** |
| After 3 months | NA | NA | NA |  | 1.20 | 0.43 – 3.36 | 0.723 |
|  |  |  |  |  |  |  |  |
| Ref: reference; 95%CI: 95% confidence interval  ^a^Model 1: adjustment on gender, age, performance status, metastases localization, center, comorbidities (kidney failure, cardiovascular disease, history of other cancer), histology, delay to treatment initiation  ^b^Model 2: adjustment on gender, age, performance status, metastases localization, center, comorbidities (kidney failure, cardiovascular disease, history of other cancer), histology, year of treatment initiation  ^c^p-value for interaction treatment with performance status <0.001  significant results are in bold | | | | | | | |

**SUPPLEMENTARY MATERIAL**

| Supplementary material S2. Characteristics of the included population according to first-line therapy after propensity score matching | | | | |
| --- | --- | --- | --- | --- |
|  | **Initial population**  *(N = 5255)* | **Chemotherapy matched group**  *(N = 169)* | **Immunotherapy**  **Matched group**  *(N = 169)* |  |
|  |  |  |  |  |
| **Patients characteristics** |  |  |  |  |
| Sex |  |  |  |  |
| Male | 3556 (67.7) | 114 (67.7) | 116 (68.6) |  |
| Female | 1699 (32.3) | 55 (32.5) | 53 (31.4) |  |
| Age at metastatic diagnosis | 63 (56 – 69) | 63 (58 – 69) | 64 (57 – 70) |  |
|  |  |  |  |  |
| **Comorbidities** |  |  |  |  |
| Smoking status |  |  |  |  |
| Active smoker | 1995 (38.0) | 68 (40.2) | 64 (37.9) |  |
| Former smoker | 2751 (52.3) | 92 (54.4) | 95 (56.2) |  |
| Non-smoker | 304 (5.8) | 9 (5.3) | 10 (5.9) |  |
| Medical history |  |  |  |  |
| COPD | 686 (13.1) | 31 (18.3) | 32 (18.9) |  |
| Autoimmune disease | 111 (2.1) | 4 (2.4) | 3 (1.8) |  |
| Diabetes mellitus | 627 (11.9) | 13 (7.7) | 21 (12.4) |  |
| Kidney failure | 83 (1.6) | 3 (1.8) | 1 (0.6) |  |
| Cardiovascular disease | 1694 (32.2) | 55 (32.5) | 52 (30.8) |  |
| Other cancer | 783 (14.9) | 24 (14.2) | 29 (17.8) |  |
|  |  |  |  |  |
| **PS ECOG** |  |  |  |  |
| 0 | 1038 (19.8) | 42 (24.9) | 44 (26.0) |  |
| 1 | 2114 (40.2) | 87 (51.5) | 85 (50.3) |  |
| 2 | 964 (18.3) | 26 (15.4) | 28 (16.6) |  |
| 3 | 342 (6.5) | 9 (5.3) | 9 (5.3) |  |
| 4 | 51 (1.0) | 5 (3.0) | 3 (1.8) |  |
|  |  |  |  |  |
| **Tumor-related informations** |  |  |  |  |
| Histological type |  |  |  |  |
| Squamous | 879 (16.7) | 30 (17.8) | 38 (22.5) |  |
| Non-squamous | 4376 (83.3) | 139 (82.2) | 131 (77.5) |  |
| M stadification |  |  |  |  |
| Mx | 782 (14.9) | 38 (22.5) | 29 (14.5) |  |
| M1a | 309 (5.9) | 4 (2.4) | 10 (5.9) |  |
| M1b | 1407 (26.8) | 44 (26.0) | 43 (25.4) |  |
| M1c | 2757 (52.5) | 83 (49.1) | 94 (55.6) |  |
| Metastasis localisation |  |  |  |  |
| Bone | 2306 (43.9) | 78 (46.2) | 71 (42.0) |  |
| Liver | 875 (16.7) | 38 (22.5) | 30 (17.8) |  |
| Brain and nervous system | 1786 (34.2) | 41 (24.3) | 40 (23.7) |  |
| Metastatic nodes | 841 (16.0) | 29 (17.9) | 34 (20.1) |  |
| Adrenal glands | 1362 (25.9) | 42 (24.9) | 56 (33.1) |  |
| Other | 1301 (24.8) | 51 (30.2) | 45 (26.6) |  |

Supplementary material S3. Main characteristics and results of observational studies of immunotherapy for lung cancer in real-world

|  | **Amrane et al.**  **(32)** | **Song et al.**  **(33)** | **Khozin et al.**  **(12)** | **Khozin et al.**  **(31)** | **Tamiya et al.**  **(34)** | **Ksienski et al.**  **(35)** | **Velcheti et al.**  **(36)** | | **Cortellini et al.**  **(37)** | **Facchinetti et al.**  **(38)** | **Frost et al.**  **(39)** | **Mountzios et al.**  **(40)** |
| --- | --- | --- | --- | --- | --- | --- | --- | --- | --- | --- | --- | --- |
|  |  |  |  |  |  |  | **EHR Cohort** | **Spotlight cohort** |  |  |  |  |
|  |  |  |  |  |  |  |  |  |  |  |  |  |
| **Number** of subjects | 108 | 39 | 5,257 | 1,344 | 213 | 190 | 423 | 188 | 1,026 | 153 | 153 | 265 |
| **Sex**, n (%) |  |  |  |  |  |  |  |  |  |  |  |  |
| Male | 70 (64.8) | 28 (71.8) | 2,819 (53.6) | 747 (55.6) | 176 (82.6) | 97 (51.1) | 228 (53.9) | 90 (47.9) | 674 (65.6) | 108 (71.0) | 90 (58.8) | 174 (65.7) |
| Female | 38 (35.2) | 11 (28.2) | 2,437 (46.4) | 597 (44.4) | 37 (17.4) | 93 (48.9) | 195 (46.1) | 98 (52.1) | 353 (34.4) | 45 (29.0) | 63 (41.2) | 91 (34.3) |
| **Age** (median and range) | 67 (37-87) | 62 (34-83) | 69 (62-76)^b^ | 69 (61-75) ^b^ | 71 (39-91) | 70 (41-91) | 72 (45-84) | 72 (46-84) | 70.2 (28-92) | 70 (38-85) | 69 (40-86) | 66.7 |
| **Histological Subtypes** |  |  |  |  |  |  |  |  |  |  |  |  |
| Non-squamous | 28 (25.9) | 18 (46.1) | 3,510 (66.8) | 872 (64.9) | 129 (60.6) | 148 (77.9) | 302 (71.4) | 129 (68.6) | 778 (75.8) | 118 (77) | 106 (69.3) | 170 (64.2) |
| Squamous | 80 (74.1) | 19 (48.7) | 1,535 (33.2) | 427 (31.8) | 55 (25.8) | 42 (22.1) | 99 (23.4) | 46 (24.5) | 248 (24.2) | 24 (16.0) | 32.9 (20.9) | 66 (24.9) |
| Adenosquamous carcinoma | - | 2 (5.1) | - | - | - | - | - | - |  | - | - | 6 (2.3) |
| Unprecised/other | - | - | - | 45 (4.3) | 29 (13.6) | - | 22 (5.2) | 13 (6.9) |  | 11 (7.0) | 15 (9.9) | 23 (8.6) |
| **ECOG PS** |  |  |  |  |  |  |  |  |  |  |  |  |
| 0 | 17 (15.8) | 17 (43.6) | 1 (<0.1) | - | 50 (23.5) | 125 (65.8) | 148 (35.0) | 80 (42.6) | 847 (82.6) | 0 | 115 (75.2) | 84 (31.9) |
| 1 | 53 (49.1) | 19 (48.7) | 385 (7.3) | - | 122 (57.3) |  | 275 (65.0) | 108 (57.4) |  | 0 |  | 132 (50.2) |
| 2 | 25 (23.1) | 3 (7.7) | 338 (6.4) | - | 32 (15.0) | 65 (34.2) | 0 | 0 | 175 (17.0) | 153 (100) | 32 (20.9) | 45 (17.1) |
| 3 | 0 | 0 | - | - | 9 (4.2) |  | 0 | 0 | 4 (0.4) | 0 | 6 (3.9) | 2 (0.8) |
| 4 | 0 | 0 | - | - | 1 (0.4) |  | 0 | 0 |  | 0 | 0 | 0 |
| Unknown | 13 (12.0) | 0 |  | - | 0 | 0 | 0 | 0 |  | 0 | 0 | 0 |
| **Smoking status** |  |  |  |  |  |  |  |  |  |  |  |  |
| Current | 34 (31.5) | 27 (69.2) | 4,679 (89.0) | - | 193 (90.6) | 67 (35.3) | 390 (92.2) | 171(91.0) | 348 (33.9) | 51 (33.3) | 125 (92.6) | 93 (35.1) |
| Former | 62 (57.4) |  |  | - |  | 109 (57.4) |  |  | 572 (55.8) | 74 (48) |  | 145 (54.7) |
| Never | 4 (3.7) | 12 (30.7) | 553 (11.0) | - | 20 (9.4) | 14 (7.4) | 33 (7.8) | 17 (9.0) | 106 (10.3) | 16 (11.0) | 10 (7.4) | 23 (8.7) |
| **TNM Stage** |  |  |  |  |  |  |  |  |  |  |  |  |
| 0 | - | - | 1 (<0.1) | - | - | - | 0 | - | 0 | 0 | 0 | 0 |
| I | - | - | 385 (7.3) | 81 (6.0) | - | - | 0 | - | 0 | 0 | 0 | 3 (1.1) |
| II | - | - | 338 (6.4) | 85 (6.3) | - | - | 0 | - | 0 | 0 | 0 | 6 (2.3) |
| IIIB | 14 (13.0) | 3 (7.7) | 1,217 (23.2) | 270 (20.1) | 38 (17.8) | - | 0 | - | 0 | 9 (6) | 29 (19.0) | 31 (11.7) |
| IIIC |  | 8 (20.5) |  |  |  | - | 0 | - | 0 |  |  |  |
| IVA | 94 (87.0) | 18 (46.2) | 3,159 (60.1) | 863 (64.2) | 144 (67.6) | 142 (74.7) | 423 (100.0) | 159 (84.6) | 1026 (100.0) | 144 (94) | 124 (81.0) | 224 (84.8) |
| IVB |  | 10 (25.6) |  |  |  |  |  |  |  |  |  |  |
| **Line of therapy (immunotherapy)** |  |  |  |  |  |  |  |  |  |  |  |  |
| First line | 108 (100.0) | 26 (66.7) | 1,329 (25.3) | 227 (16.9) | 213 (100.0) | 141 (74.2) | 423 (100.0) | 159 (100.0) | 1026 (100.0) | 153 (100.0) | 153 (100.0) | 265 (100.0) |
| >1 | 0 | 13 (13.3) | 3,927 (74.7) | 1117 (83.1) | 0 | 49 (25.8) | 0 | 0 | 0 | 0 | 0 | 0 |
| **PD-L1 status** |  |  |  |  |  |  |  |  |  |  |  |  |
| Positive | 108 (100.0) | 16 (41.0) | 1219 (23.2) | 55 (49.1) | 213 (100.0) | 190 (100.0) | 423 (100.0) | 188 (100.0) | 1026 (100.0) | 153 (100.0) | 153 (100.0) | 265 (100.0) |
| <1% | 0 | - | 312 (20.8) | - | 0 |  | 0 | 0 |  | 0 | 0 | 0 |
| 1-49% | 0 | - | 285 (19.0) | - | 0 | 14 (7.4) | 0 | 0 |  | 0 | 0 |  |
| >50% | 108 (100.0) | - | 622 (41.4) | - | 213 (100.0) | 176 (92.6) | 423 (100.0) | 188 (100.0) | 1026 (100.0) | 153 (100.0) | 153 (100.0) | 265 (100.0) |
| 50-74% | - | - | - | - | 97 (45.5) | - |  |  |  | 71 (46) | 104 (68,0) ^c^ | 0 |
| 75-90% | - | - | - | - | 47 (22.1) | - |  |  |  | 29 (19) | 24 (15.3) ^d^ | 0 |
| 90-100% | - | - | - | - | 69 (32.4) | - | 186 (44.0) | 74 (39.3) |  | 23 (15) | 25 (16.3) | 0 |
| Negative/unknown | - | 23 (59.0) | - | 37 (33.0) | - | - | - | - |  | - | - |  |
| Unknown/missing | - | - | 283 (18.8) | 20 (17.9) | - | - | - | - |  | - | - |  |
| **Metastasis** |  |  |  |  |  |  |  |  |  |  |  |  |
| Brain metastasis | 19 (17.6) | - | - | - | - | 26 (13.7) | 48 (11.3) | 23 (12.2) | 181 (17.6) | - | 32 (20.9) | 47 (17.7) |
| Liver metastasis | - | - | - | - | 28 (13.1) | 17 (8.9) |  | - | 158 (15.4) | - | 15 (9.8) | 35 (13.2) |
| **Outcomes as first-line** |  |  |  |  |  |  |  |  |  |  |  |  |
| **OS**, months (95% CI) | 15.2 (13.9-NR) | Not reached | 10.75 (9.61-11.7) | 8.3 (7.4-10.6) | 17.8 (17.8- NR) | 24.3 (9.7- NR) | 18.9 (14.9-25.5) | 19.1 (12.6 – NR) | 17.2 (15.3 -22.3) | 3.0 (2.4 – 3.5) | 22.0 (15.4 – 28.6) | 22.5 |
| **PFS**, months (95% CI) | 10.1 (8.8-11.4) | 25.5 (6.8-44.1) ^a^ | 4.26 (3.8-4.79) | - | 8.3 (6.0-10.7) | - | - | 6.8 (5.3-8.1) | 7.9 (6.9-9.5) | 2.4 (1.6-2.5) | 8.2 (5.1 -11.4) | 10.4 |
| ^a^ For all the population (not only as first line)  ^b^ Inter-quartile range  ^c^ PD-L1 expression 50-79%  ^d^ PD-L1 expression 80-89%  ECOG PS : Eastern Cooperative Oncology Group Performance Status | | | | | | | | |  |  |  |  |
